# Digested wheat gluten inhibits binding between leptin and its receptor

**DOI:** 10.1186/s12858-015-0032-y

**Published:** 2015-01-20

**Authors:** Tommy Jönsson, Ashfaque A Memon, Kristina Sundquist, Jan Sundquist, Stefan Olsson, Amarnadh Nalla, Mikael Bauer, Sara Linse

**Affiliations:** Center for Primary Health Care Research, Lund University/Region Skåne, Skåne University Hospital, Malmö, Sweden; Department of Plant and Environmental Science, University of Copenhagen, DK-1871 Frederiksberg C, Denmark; Institute of Biomedical Sciences, Faculty of Health and Medical Sciences, University of Copenhagen, DK-2200 Copenhagen, Denmark; The Danish Diabetes Academy, Odense University Hospital, Odense, Denmark; Department of Biochemistry and Structural Biology, Lund University, Lund, Sweden

**Keywords:** Gluten, Leptin, Leptin resistance, Obesity

## Abstract

**Background:**

Leptin resistance is considered a primary risk factor for obesity. It has been hypothesized that dietary cereal grain protein could cause leptin resistance by preventing leptin from binding to its receptor. Non-degraded dietary wheat protein has been found in human serum at a mean level of 41 ng/mL. Here, we report our findings from testing whether enzymatically digested gluten from wheat prevents leptin from binding to the leptin receptor *in vitro*.

Gluten from wheat was digested with pepsin and trypsin under physiological conditions. Pepsin and trypsin activity was removed from the gluten digest with a 10 kDa spin-filter or by heat treatment at 100°C for 30 min. Binding to the leptin receptor of leptin mixed with gluten digest at a series of concentrations was measured using surface plasmon resonance technology.

**Results:**

Binding of the gluten digest to the leptin receptor was not detected. Spin-filtered gluten digest inhibited binding of leptin to the leptin receptor, with 50% inhibition at a gluten digest concentration of ~10 ng/mL. Heat-treated gluten digest did not inhibit leptin binding.

**Conclusions:**

Digested wheat gluten inhibits binding of leptin to the leptin receptor, with half-maximal inhibition at 10 ng/mL. The inhibition is significant at clinically relevant concentrations and could therefore serve as a novel pathway to investigate to understand the molecular basis of leptin resistance, obesity and associated disorders.

## Background

Leptin is a 16-kDa polypeptide secreted by white adipose tissue into the circulation, as recently reviewed by Zhou *et al.* [1]. Circulating leptin levels are proportional to body fat mass and fluctuate in accordance with changes in nutritional states. The leptin concentration serves as a key adiposity signal for the brain, where leptin binds to and activates the leptin receptor. Leptin is important in regulating satiety, weight and energy homeostasis. Most obese patients have high levels of circulating leptin, indicating an acquired state of leptin resistance, defined by the reduced ability of leptin to suppress appetite and weight gain [2]. Leptin resistance is considered a primary risk factor for the pathogenesis of overweight and obesity [2], which in turn is closely associated with various metabolic disorders including dyslipidemia, cardiovascular disease, stroke, insulin resistance and type 2 diabetes. Several mechanisms have been proposed to explain leptin resistance, including impaired leptin transport, leptin signaling and leptin-targeted neural circuits [2].

It has been hypothesized that dietary cereal grain protein could cause leptin resistance by preventing leptin from binding to the leptin receptor [3]. Briefly, the hypothesis rests on the following propositions: _(I)_ The global pattern of varying prevalence of diseases of affluence, such as obesity, cardiovascular disease and diabetes, suggests that some environmental factor specific to agrarian societies could initiate these diseases; _(II)_ A diet based on cereal grain could be such an environmental factor; _(III)_ Leptin resistance is also associated with diseases of affluence and could be a sign of insufficient adaptation to a diet based on cereal grain; _(IV)_ Cereal grain proteins have sufficient properties (i.e. they are unique, are present in human food, are heat-stable, are resistant to gastro-intestinal breakdown, enter the human circulation, and bind to cell surfaces and receptors) to cause leptin resistance by inhibiting binding of leptin to the leptin receptor. The hypothesis is supported by a recent study on human genetic adaptation by Segurel *et al*., which showed positive selection of protective variants of the leptin receptor with regard to type 2 diabetes from the Neolithic period onward [4]. This indicates the onset of evolutionary pressure on the leptin receptor when significant amounts of cereal grain were adopted as food. Support also comes from animal studies, which show that dietary components can induce leptin resistance in rats in relatively short periods of time at normal body weight and leptin levels [5]. Dall *et al*. demonstrated that cereal grain protein in the form of digested wheat gliadin caused a 20% increase in weight gain when injected into non-obese diabetic (NOD) mice in a pre-diabetic state [6], possibly indicating the induction of leptin resistance. Dall *et al*. also demonstrated that digested wheat gliadin caused a dose-dependent increase in insulin secretion when incubated with rat insulinoma cells and rat islets, which was possibly caused by inhibition of current through ATP-sensitive K+ (K_ATP_) channels [6]. These findings could be indirect evidence that digested wheat gliadin actually inhibits leptin binding, since leptin was previously reported to cause the opposite effect in insulinoma cells [7]. Further support comes from human clinical studies, in which El-Shebini *et al*. found that indices of leptin resistance are improved by replacing bread with vegetables in otherwise similar hypocaloric diets and with similar weight loss [8]. Another study by Ryberg *et al*. on the effects of a diet without cereal grains also showed significant effects on leptin [9]. Our previous dietary intervention study comparing effects of diets with and without cereal grains showed a strong correlation between relative change in leptin and cereal grain intake [10].

In this study, we tested the last proposition of the above hypothesis, which is that cereal grain proteins inhibit leptin binding. Cereal grain proteins have already been reported to bind to a receptor by Lammers *et al*., who found that cereal grain peptides derived from enzymatic digestion of wheat gliadin with the gut enzymes pepsin and trypsin under physiological conditions bind to the chemokine receptor CXCR3 expressed in mouse and human intestinal epithelia and laminae propriae, leading to zonulin release and increased intestinal permeability [11]. Kamikubo *et al*. reported that wheat germ agglutinin binds to the leptin receptor *in vitro* and inhibits binding of leptin to the leptin receptor [12]. Wheat germ agglutinin is found in common wheat flour but not in human blood [13]. We chose to examine cereal grain protein from wheat, which is the main source of vegetable protein in human food. The main protein component of wheat is gluten, which is the cohesive and elastic mass that remains after starch has been removed from cereal grain flour by rinsing with water. More specifically, wheat gluten is a composite of several kinds of proteins, such as gliadins (molecular weight ~30 kDa) and glutenins (molecular weight ~30-90 kDa). Gluten intake has increased greatly over the last hundred years and has accelerated during the last few decades [14,15]. This increase is largely due to breeding of gluten-rich cereal grain varieties and most recently by the use of extra gluten in baking and food processing to make dough easier to work and bread fluffier [15]. Soares *et al*. found that a gluten-free diet reduces leptin, adiposity, inflammation and insulin resistance in mice despite a similar energy intake [16]. Chirdo *et al*. reported the presence of non-degraded wheat gliadin in human serum (at a mean level of 41 ng/mL) [17], as previously also reported for other dietary proteins by Husby *et al*. [18,19].

Here, we used surface plasmon resonance (SPR) technology to monitor the interaction between leptin and the leptin receptor and its inhibition by enzymatically digested gluten from wheat.

## Methods

### Digestion of gluten

To mimic physiological conditions in the human intestine, gluten from wheat was digested according to the protocol of De Ritis *et al*. [20] with slight modifications. 100 g of gluten from wheat (Sigma-Aldrich: G5004) was digested in 1 L of 0.2 N HCl (pH 1.8) containing 2 g of pepsin (Sigma-Aldrich: P6887) at 37°C for 2 hours. The pH was checked periodically and adjusted to 1.8 with HC1 or NaOH as necessary. The pH was then adjusted to 8.0 with 2 N NaOH. The pepsin-digested gluten was further digested by addition of 2 g of trypsin (Sigma-Aldrich: T4799). The resulting pepsin- and trypsin-digested gluten was vigorously stirred at 37°C for 4 h. The pH was checked periodically and adjusted to pH 8.0 with HC1 or NaOH as necessary.

### Pepsin and trypsin removal

Pepsin (molecular weight ~40 kDa) and trypsin (molecular weight ~25 kDa) were removed from the gluten digest by either spin-filtering through a 10 kDa filter or heat-treatment at 100°C for 30 min followed by centrifugation at 13000 g for 10 min. The gluten digest concentration after filtering or centrifugation was determined from the absorbance at 280 nm, assuming an absorbance of 1 at 1 mg/mL. To check whether any pepsin or trypsin activity remained, 1.8 μg/mL leptin (recombinant human leptin, R&D Systems) was incubated in gluten digest (spin-filtered only or spin-filtered and heat-treated) for 1 h or 24 h at 37°C. The samples were resolved by SDS PAGE (Invitrogen) and were blotted using the iBlot® Gel Transfer Stacks, PVDF, mini kit (Invitrogen) according to the manufacturer’s instructions. After washing, the blots were incubated with anti-leptin HRP-conjugated antibody (HyTest Ltd, cat. # 2LE1C) at 4°C overnight. The antibody was used at a 1:1000 dilution relative to the stock concentration in the product (HyTest Ltd, cat. # 2LE1C). Immunoreactive bands were detected using ECL reagents (GE Healthcare Life Sciences).

### SPR studies

All SPR experiments were performed using the Biacore 3000 system (Biacore AB, Uppsala, Sweden) and Sensor Chips coated with carboxylated dextran (CM5, GE Healthcare). The flow rate was 10 μL/min throughout immobilization and all experiments. Immobilization of human leptin receptor/Fc chimera (R&D Systems 389-LR) was performed using amine coupling with HBS-EP running buffer (10 mM HEPES, 3.4 mM EDTA, 150 mM NaCl, 0.005% Tween 20, pH 7.4). The Sensor Chip surface was activated by injecting a solution of 0.05 M NHS and 0.2 M EDC in water (mixed just prior to injection) for 7 min, followed by a short buffer rinse. Coupling was achieved by injecting 100 μl of human leptin receptor/Fc chimera in 10 mM sodium acetate buffer (pH 5.0) into three flow cells at concentrations of 1, 3 and 10 μg/mL. One channel received no receptor to serve as a blank control. All four flow cells were blocked by injection of 70 μl of 1 M ethanolamine (pH 8.5), followed by buffer flow for at least 2 h. Association of human leptin (R&D Systems: 398-LP) was monitored by injection of 30 nM leptin for 10 min, and its dissociation was monitored by buffer flow for up to 15 h. The instrument reports the amount bound per surface area in RU (1 RU = 1 ng/mm^2^), as derived from the angle of minimum total internal reflection of the incident light. The experiment was repeated three times for a series of 30 nM leptin samples with increasing concentrations of gluten digest (heat-treated or spin-filtered) ranging from 0.9 ng/mL to 450 μg/mL. In these experiments, the association of leptin was monitored for 10 min and dissociation for 5 min. Dissociation was followed by regeneration by injection of 25 mM glycine/HCl (pH 2.5) for 7 min.

The dissociation phase data were fitted using a single exponential decay:

1 $$ \mathrm{I} = {\mathrm{I}}_0 \cdot p {\mathrm{e}}^{\hbox{-} \mathrm{koff}\cdotp\ \mathrm{t}} + {\mathrm{R}}_{\infty } $$

The value obtained for k^off^ was then used during fitting of the association phase data to obtain an estimate of k^on^ with the following equation:

2 $$ \mathrm{I} = {\mathrm{R}}_{\max } \cdot p \mathrm{c}\cdotp\ {\mathrm{k}}^{\mathrm{on}}\cdotp \left({1\ \hbox{-}\ \mathrm{e}}^{\hbox{-} \left(\mathrm{c}\cdotp\ \mathrm{k}\mathrm{o}\mathrm{n} + \mathrm{ko}\mathrm{ff}\right)\cdotp\ \mathrm{t}}\right)/\left(\mathrm{c}\cdotp\ {\mathrm{k}}^{\mathrm{on}} + {\mathrm{k}}^{\mathrm{off}}\right) + {\mathrm{R}}_{\infty } $$

The inhibition data were analyzed by finding the plateau value at the end of each injection of leptin alone or leptin plus gluten digest.

## Results

### Pepsin and trypsin removal

Western blotting showed remaining leptin bands for all gluten digests after both 1 and 24 h. This shows that spin-filtering and heat treatment removed all protease activity in the gluten digest since leptin would otherwise have been degraded.

### SPR studies

**Figure 1:**
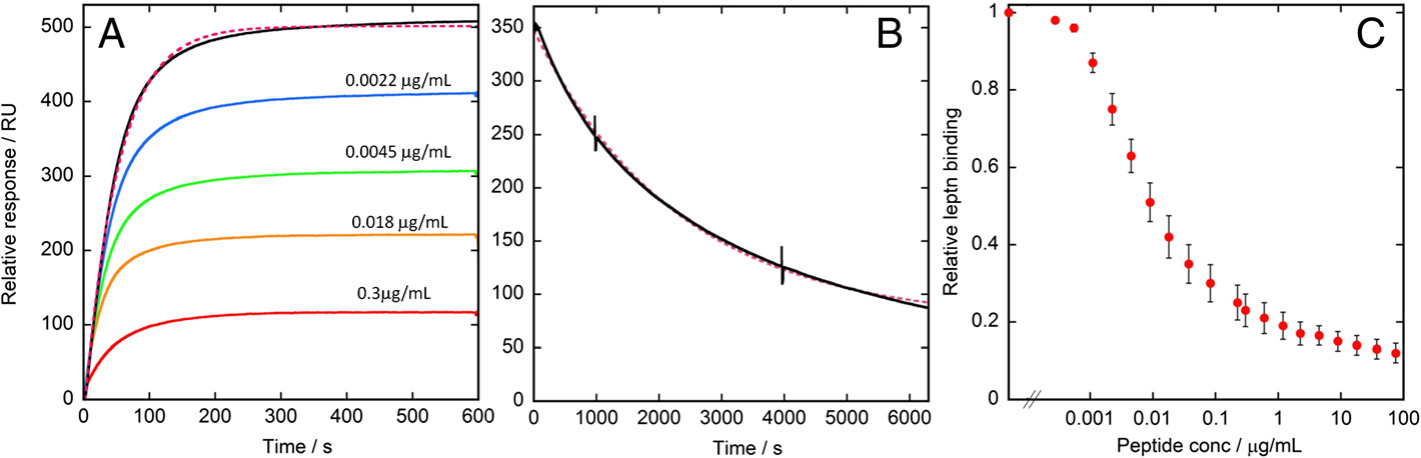
**SPR analysis of binding of leptin to the leptin receptor and its inhibition by the gluten digest.** **A)** Examples of sensorgrams recorded during the injection of 30 nM leptin alone (black) or in the presence of 0.0022 (blue), 0.0045 (green), 0.018 (orange) or 0.3 (red) μg/mL gluten digest over a Sensor Chip with immobilized leptin receptor-Fc chimera. The pink dashed line is a fit to the black line using equation 2 (see Methods). **B)** Example of a sensorgram recorded during buffer flow after complete injection of 30 nM leptin (black). The pink dashed line is a fit to the data using equation 1 (see Methods). The two vertical lines occur during the extended time dissociation when the machine switches between its two pumps. **C)** Plot of relative intensity at the final leptin binding plateau during injection of 30 nM leptin versus gluten digest concentration. The error bars represent the standard deviation of three measurements.

Attempts to monitor binding of gluten digest to the leptin receptor suffered from too low a signal-to-noise ratio and were therefore inconclusive. Instead, we studied binding to the leptin receptor of leptin alone and leptin mixed with gluten digest at a series of concentrations. Leptin alone was found to interact with the leptin receptor with high affinity (k^off^ = 4.3^.^10^-4^ s^-1^, k^on^ = 6.4^.^10^5^ M^-1^ s^-1^, K_D_ = 0.6 nM, Figure 1A,B), in line with earlier findings [21]. The heat-treated gluten digest did not inhibit binding of leptin to the leptin receptor. However, the spin-filtered gluten digest reduced binding of leptin to the leptin receptor in a concentration-dependent manner. Examples of SPR sensorgrams for a few concentrations of spin-filtered gluten digest are shown in Figure 1A, with the relative concentration of bound leptin at the plateau value shown for all concentrations in Figure 1C (see below). While experiments with non-purified proteolytic gluten digest would suffer from potential digestion of the receptor on the chip, we here evaluated two methods for protease inactivation/removal. While heat treatment followed by centrifugation seems to remove all protease activity (leptin bound with an undiminished signal during repeated injections), it also removed all leptin-inhibiting activity from the gluten digest. The use of molecular weight filters was successful and seems to remove all protease activity while leaving leptin-inhibiting activity intact in the remaining filtrate. The final leptin binding plateau value obtained in the absence or presence of the filtrated gluten digest is plotted versus the logarithm of the gluten digest concentration (Figure 1C). The gluten digest was found to dose-dependently inhibit binding of leptin to the leptin receptor. A sigmoidal curve was obtained, with half-maximal inhibition of leptin binding at a gluten digest concentration of ~10 ng/mL.

## Discussion

### Key findings

Digested wheat gluten inhibits binding of leptin to the leptin receptor.

### Possible mechanisms

No binding of gluten digest to the leptin receptor was detected. This may indicate that digested gluten was too small to detect when bound to the leptin receptor or that digested gluten instead bound to leptin. Regardless, the gluten digest may inhibit leptin binding directly by obstructing the binding site on leptin or the leptin receptor, or indirectly by causing a conformational change in leptin or the leptin receptor that disturbs their ability to bind one another.

### Comparison with findings from other studies

Lammers *et al*. showed that gluten digest caused concentration-dependent displacement of the CXCR3 receptor ligand, with 50% ligand displacement with a gluten digest concentration of 1 mg/mL [11]. The much lower gluten digest concentration of 10 ng/mL needed in our study for half-maximal inhibition of leptin binding could be due to sensitivity differences in the studies’ respective binding model. It could also be due to concentration and/or activity differences between the inhibiting substances in the respective gluten digests.

### Limitations of the present study

This is an *in vitro* study and more research has to be performed to clarify the possible clinical relevance of our observations. Also, the study examined effects of wheat gluten, thus leaving other wheat proteins and all other cereal grain proteins for future studies. Furthermore, protease activity was removed from the gluten digest by spin-filtering through a 10 kDa filter. This will have removed larger possibly active substances from the gluten digest. Such substances can be examined in future studies.

### Research and clinical implications

The concentrations at which digested wheat gluten inhibited leptin in our study are in the same range as the concentrations previously reported for gliadin and other dietary proteins in human serum [17-19], thus making cereal grain proteins clinically relevant as a possible cause of leptin resistance and obesity. Our findings warrant further research, not only on the effects of proteins from wheat and other cereal grains on leptin signalling, but also on the effects of other dietary proteins on other receptors, structures and functions in the body.

To assess the clinical implications of the study results, we should consider previous findings on the relationship between serum leptin and body fat mass in humans, which was found to be a strong linear or quadratic correlation (R = 0.86, P < 0.0001 for the linear correlation and R = 0.85, P < 0.001 for the quadratic correlation), as measured by underwater weighing or bioelectric impedance analysis [22,23]. Also, another study showed that there was a linear relationship between serum leptin and cerebrospinal fluid leptin in lean individuals (R = 0.41, P < 0.05) [24]. Such correlations are of course not certain indications of a causal connection and most certainly oversimplify the mechanisms causing obesity. However, if there were a causal linear relationship between leptin level and body fat mass, a tentative 50% reduction in binding of leptin to the leptin receptor due to continual intake of cereal grain proteins would lead to a doubling of body fat mass. Furthermore, for an adult with 20% body fat mass, a doubling of body fat mass would increase body mass index (BMI) by 20%. This is the difference between current mean BMI among Swedish adults of ~25 kg/m^2^ and a healthier ~21 kg/m^2^. A corresponding BMI improvement would probably also reduce obesity-associated metabolic disorders such as dyslipidemia, cardiovascular disease, stroke, insulin resistance and type 2 diabetes in the population.

## Conclusions

Digested wheat gluten inhibits binding of leptin to the leptin receptor, with half-maximal inhibition at 10 ng/mL. The inhibition is significant at clinically relevant concentrations and could therefore serve as a new pathway to investigate to understand the molecular basis of leptin resistance, obesity and associated disorders.
